# Oncolytic Vaccinia Virus Expressing *Aphrocallistes vastus* Lectin as a Cancer Therapeutic Agent

**DOI:** 10.3390/md17060363

**Published:** 2019-06-19

**Authors:** Tao Wu, Yulin Xiang, Tingting Liu, Xue Wang, Xiaoyuan Ren, Ting Ye, Gongchu Li

**Affiliations:** Zhejiang Sci-Tech University Hangzhou Gongchu Joint Institute of Biomedicine, College of Life Sciences and Medicine, Zhejiang Sci-Tech University, Hangzhou 310018, China; wutao0920@163.com (T.W.); q522329467@163.com (Y.X.); m13617965853@163.com (T.L.); wx18815610822@163.com (X.W.); imrenxy@163.com (X.R.)

**Keywords:** *Aphrocallistes vastus* lectin, oncolytic vaccinia virus, ERK

## Abstract

Lectins display a variety of biological functions including insecticidal, antimicrobial, as well as antitumor activities. In this report, a gene encoding *Aphrocallistes vastus* lectin (AVL), a C-type lectin, was inserted into an oncolytic vaccinia virus vector (oncoVV) to form a recombinant virus oncoVV-AVL, which showed significant in vitro antiproliferative activity in a variety of cancer cell lines. Further investigations revealed that oncoVV-AVL replicated faster than oncoVV significantly in cancer cells. Intracellular signaling elements including NF-κB2, NIK, as well as ERK were determined to be altered by oncoVV-AVL. Virus replication upregulated by AVL was completely dependent on ERK activity. Furthermore, in vivo studies showed that oncoVV-AVL elicited significant antitumor effect in colorectal cancer and liver cancer mouse models. Our study might provide insights into a novel way of the utilization of marine lectin AVL in oncolytic viral therapies.

## 1. Introduction

Lectins, as a class of specific glycosyl-binding glycoproteins, preferentially recognize and bind carbohydrate complexes [1,2]. Since the first discovery in 1888 [3], hundreds of lectins have been isolated and characterized. Lectins were obtained from microorganisms, animals, and plants. Animal and plant lectins lack primary structural homology [4,5,6], but they have demonstrated similar carbohydrates-binding activities. Animal lectins are categorized into C-type lectins, Galectins, P-type lectins, L-Type Lectins, R-Type Lectins, and I-type lectins [7]. In past decades, lectins have been developed to form a variety of biological techniques, such as lectin array, lectin blot, as well as lectin-based chromatography, to analyze glycofiles and biomarkers for various cancers, including ovarian cancer [8], pancreatic cancer [9], prostate cancer [10], aggressive breast cancer [11,12], and liver cancer [13]. In addition, lectins such as *Maackia amurensis* seed lectin [14], Concanavalin A [15], *Fenneropenaeus indicus* hemolymph fucose binding lectin [16], *Polygonatum cyrtonema* lectin [17], as well as MytiLec [18,19,20] were shown to be cytotoxic to cancer cells through inducing apoptosis or autophagy. Furthermore, various lectins delivered through viral vectors elicited anticancer effect in vitro and in vivo [21,22,23,24,25,26].

Sponges, which are known to be one of the oldest living marine organisms, produce different types of biological molecules, such as okadaic acid and halichondrin B [27]. Halichondrin B from the Japanese black sponge *Halichondria okadai* as an effective anticancer drug [28], can modulate microtubule dynamics. Many types of lectins are also produced in various species of sponges, including galectins, N-acetylamino-carbohydrate-specific lectin, c-type lectin, and so on [29,30]. The *Aphrocallistes vastus* lectin (AVL) is a Ca2+-dependent lectin and inhibited by bird’s nest glycoprotein and D-galactose. AVL shows the highest similarity to C-type lectins from higher metazoan phyla [31]. Here, we addressed C-type lectin from *Aphrocallistes vastus* with biological activities in enhancing the replication of oncolytic vaccinia virus and its therapeutic effect for cancers.

Oncolytic viruses have the advantage of high killing effects without drug resistance. Since the first report of a thymidine kinase (TK) deleted herpes simplex virus (HSV) in cancer treatment [32], more than 10 families of oncolytic viruses have entered clinical trials, including adenovirus, coxsackie virus, herpes simplex virus, measles virus, new castle disease virus, parvovirus, poliovirus, reovirus, and vesicular stomatitis virus [33]. Among them, vaccinia virus (VV) has the longest history of use in humans. VV can be used as replicating vectors harboring therapeutic genes to directly lyse tumor cells or as cancer vaccines to stimulate antitumor immunity. JX-594 is an oncolytic vaccinia virus carrying a human granulocyte-macrophage colony-stimulating factor (GM-CSF) gene with the TK gene deletion [34]. Up to now, JX-594 has been advanced to clinical phase III for the treatment of advanced hepatocellular carcinoma (HCC) and clinical phase I trial for renal cell carcinoma [35]. In addition, there are other oncolytic vaccinia viruses that have entered clinical trials, including GL-ONC1 and vvDD-CDSR [36].

Previously, the harboring of a gene encoding *Tachypleus tridentatus* lectin (TTL) was shown to enhance the therapeutic effect of oncolytic vaccinia virus in a hepatocellular carcinoma mouse model [21], suggesting that harboring lectin genes may enhance the therapeutic effect of oncolytic viruses. At present, the therapeutic effect of lectins was determined in animal models and in cancer cell lines. However, there are still no lectins entering clinical trials. In this study, a gene encoding AVL was inserted into an oncolytic vaccinia virus (oncoVV) vector, which is deficient of TK gene for cancer specific replication [37], forming a recombinant virus oncoVV-AVL. The antitumor effect of oncoVV-AVL and the underlying mechanisms were analyzed.

## 2. Results

### 2.1. AVL Expression Through a Non-Replicating Adenovirus Showed Cytotoxicity in a Variety of Cancer Cells

**Figure 1:**
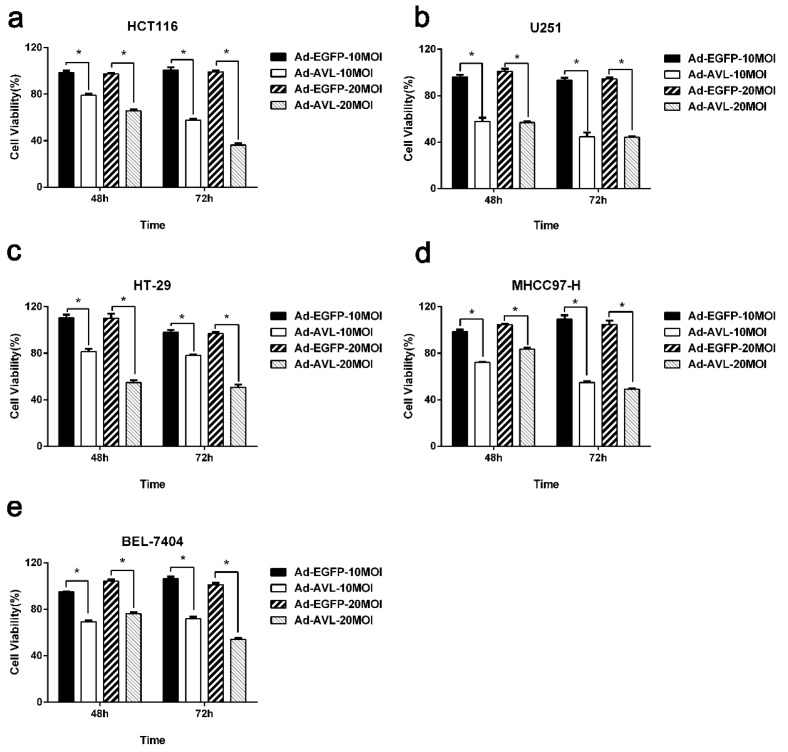
The cytotoxicity of Ad-AVL in various tumor cells. The cytotoxicity of Ad-AVL was measured by MTT assay in HCT116 cells (**a**), U251 cells (**b**), HT-29 cells (**c**), MHCC-97-H cells (**d**), and BEL-7404 cells (**e**). Ad-EGFP served as a control. Data were expressed as the mean ± SEM from at least three separate experiments. (* *p* < 0.05).

In previous studies, adenoviruses (Ad) harboring lectins had shown significant cytotoxicity to a variety of cancer cells [22,23,24,25]. To investigate the cytotoxicity of exogenously expressed AVL, Ad-AVL, a non-replicating adenovirus carrying an AVL gene, was assessed by MTT assay in colorectal cancer cell line HCT116, glioma cell line U251, hepatocellular carcinoma cell lines BEL-7404, and MHCC97-H, as well as colon cancer cell line HT-29. Ad-EGFP was used as a control. As shown in the Figure 1, the cell viability after the treatment of Ad-AVL in tumor cells was obviously lower than that of Ad-EGFP. The results indicated that exogenous AVL expression elicited cytotoxicity in various cancer cells.

### 2.2. AVL Significantly Enhanced the Antiproliferative Effect of Oncolytic Vaccinia Virus

**Figure 2:**
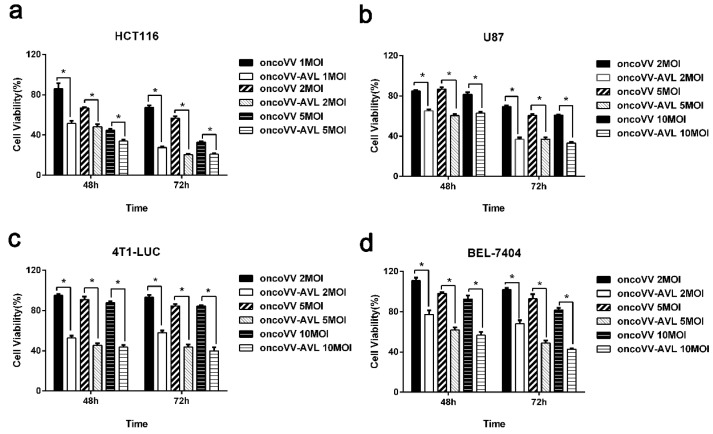
The antiproliferative effect of oncoVV-AVL in cancer cells. The cell viability was measured by MTT assay in HCT116 cells (**a**), U87 cells (**b**), 4T1-LUC (**c**), and BEL-7404 cells (**d**). OncoVV was used as a control virus. Data were expressed as the mean ± SEM from at least three separate experiments. (* *p* < 0.05).

In order to determine the antiproliferative efficacy of oncolytic vaccinia virus oncoVV-AVL, the viability of various cell lines treated with oncoVV-AVL was investigated. The empty vector oncoVV served as the control. After virus infection at different MOIs for 48 h and 72 h, the viability of the cell lines (HCT116, U87, BEL-7404 and 4T1-LUC) was measured by MTT assay. As shown in Figure 2a, the cell viability of the oncoVV-AVL group was obviously lower than that of oncoVV in HCT116 cells, showing a time and dosage dependent manner. As shown in Figure 2b–d, similar results were yielded in U87, 4T1-LUC, and BEL-7404 cells. Taken together, the results indicated that AVL promoted the antiproliferative efficacy of oncolytic vaccinia virus in cancer cells.

### 2.3. AVL Improve the Replication Ability of Oncolytic Vaccinia Virus

**Figure 3:**
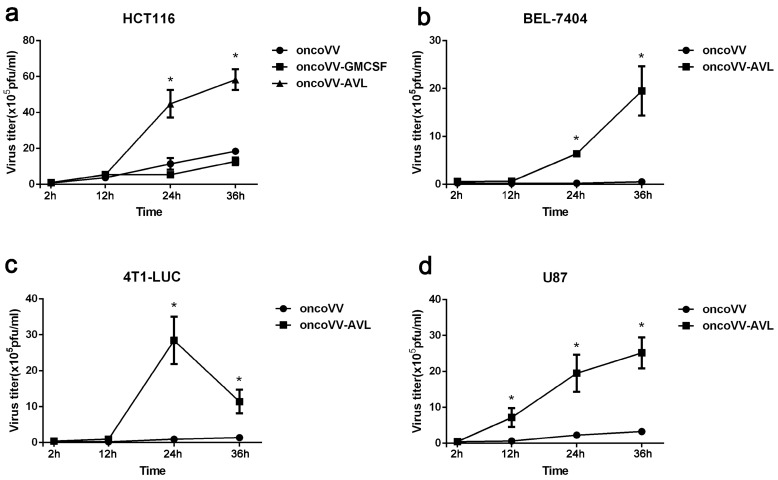
oncoVV-AVL replication in multiple tumor cell lines. The replication of oncoVV-AVL in HCT116 cells (**a**), BEL-7404 cells (**b**), 4T1-LUC cells (**c**), and U87 cells (**d**). Viral replication was determined by TCID_50_ assay. Data were expressed as the mean ± SEM from at least three separate experiments. (* *p* < 0.05).

In order to investigate the underlying mechanism of the enhanced antiproliferative effect of oncoVV-AVL, the replication ability of oncoVV-AVL, oncoVV, and oncoVV-GM-CSF was tested through TCID_50_ method. As shown, oncoVV-AVL replicated significantly faster than control viruses in HCT116 cells, BEL-7404 cells, 4T1-LUC cells, and U87 cells (Figure 3). The results show that oncolytic vaccinia virus harboring AVL gene significantly improved the replication ability in various cancer cells.

### 2.4. OncoVV-AVL Altered ERK and NF-κB Signaling Pathways in Cancer Cells

**Figure 4:**
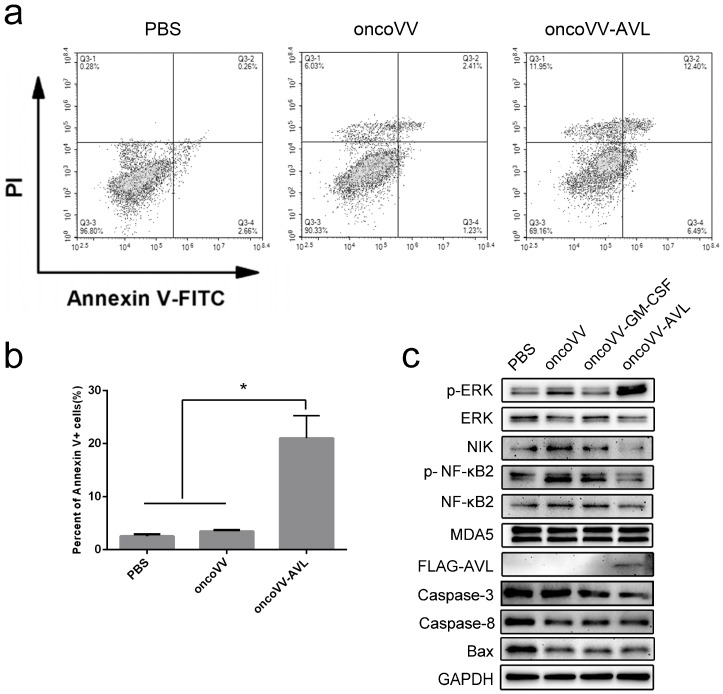
oncoVV-AVL induced apoptosis and altered various intracellular signaling pathways in HCT116 cells. (**a**) HCT116 cells were treated with oncoVV or oncoVV-AVL at 2 MOI as well as PBS control for 24 h. Cells were stained with Annexin V-FITC and PI followed by analysis under a flow cytometer; (**b**) the percent of Annexin V-positive cells from three repeats was shown as mean ± SEM (* *p* < 0.05). (**c**) The levels of p-ERK, ERK, MDA5, NF-κB2, p-NF-κB2, NIK, Caspase-3, Caspase8, Bax and FLAG tagged AVL was detected by Western blot. GAPDH served as a loading control.

In order to explore the underlying molecular mechanism of the enhanced cytotoxicity and replication ability of oncoVV-AVL, apoptosis analysis and intracellular signaling elements examination were performed in HCT116 cells. Cells were treated with oncoVV, oncoVV-AVL, or PBS followed by staining with Annexin V-FITC and propidium iodide (PI), a common method for apoptotic cell staining, and analysis under a flow cytometer. As shown in Figure 4a, oncoVV-AVL induced a higher percent of Annexin V^+^/PI^−^ and Annexin V^+^/PI^+^ cells, as compared to the cells treated with either PBS or oncoVV. Significant differences achieved from three repeats are shown in Figure 4b. Results indicate that oncoVV-AVL induced apoptosis in HCT116 cells. The Western blot results were shown in Figure 4c, and densitometry analysis was shown in Supplementary Figure S1. Melanoma differentiation-associated protein 5 (MDA5), a DDX58 -like receptor, is a dsRNA helicase [38]. Previous studies have shown that MDA5 inhibited the replication of encephalomyocarditis virus (EMCV) and vesicular stomatitis virus (VSV) [39]. Our results showed that there was no significant difference of MDA5 levels among oncoVV-AVL and other treatments, suggesting that oncoVV-AVL did not alter the MDA5 pathway in HCT116 cells.

Caspase 3, a member of the caspase family, plays a key role in most apoptotic signaling pathways. The appearance of apoptosis leads to the cleavage and activation of Caspase-3 by various proteolytic enzymes [40]. Our results showed that the level of Caspase-3 of oncoVV-AVL treatment was obviously lower than that of oncoVV and PBS, indicating that oncoVV-AVL stimulated caspase cleavage and enhanced apoptotic effect. We further analyzed the effect of oncoVV-AVL treatment on caspase 8 and proapoptotic factor Bax. Our data showed that all three vaccinia viruses slightly downregulated caspase 8 and Bax as compared to PBS control, but no obvious differences were observed among these viruses, indicating that AVL harboring did not affect caspase 8 and Bax.

NIK (NF-κB-inducing kinase), as a kinase of NF-κB, is a member of MAP kinase kinase kinase (MAP3K) [41]. NF-κB2 is a transcription factor in the NF-κB family and plays an important role in NF-κB-mediated inflammation and immunity [42]. In this study, oncoVV upregulated NIK level, which was downregulated by oncoVV-AVL. Furthermore, oncoVV-AVL significantly suppressed the NF-κB2 phosphorylation as compared to control viruses. The results suggested that AVL might enhance viral cytotoxicity in cancer cells by downregulating NF-κB signaling pathway.

It has been reported that extracellular signal-regulated kinase (ERK) was required for virus replication and played a critical role in transmitting signals from surface receptors to the nucleus [43]. In our data, oncoVV-AVL significantly induced ERK phosphorylation as compared to oncoVV and oncoVV-GM-CSF, suggesting that AVL might enhance viral replication by activating ERK.

### 2.5. The OncoVV Replication Upregulated by AVL Was Completely Dependent on ERK Activation

**Figure 5:**
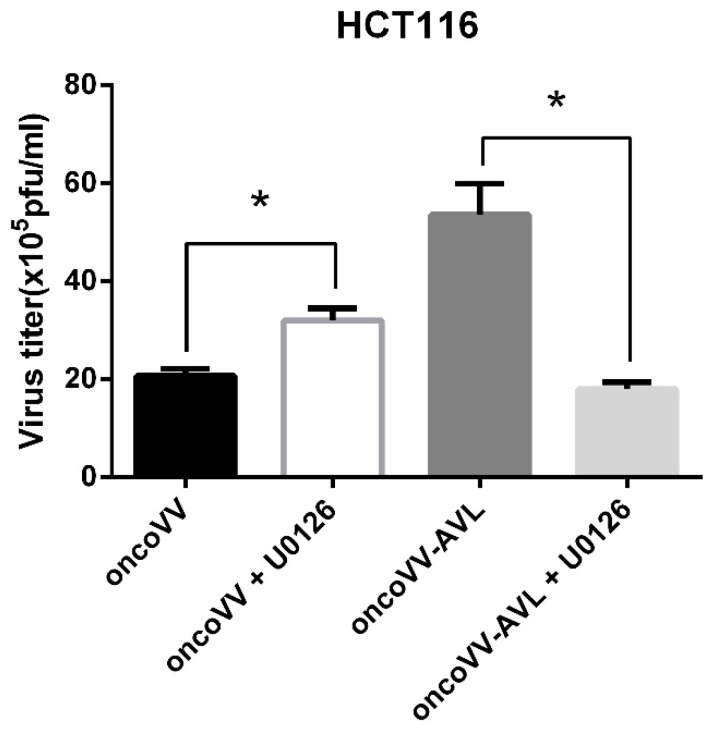
The virus titers of oncoVV and oncoVV-AVL affected by U0126 in HCT116 cells. OncoVV or oncoVV-AVL at 5MOI was used to treat HCT116 cells for 24 h in combination with 10 μM of U0126. Virus titers were measured by TCID_50_ assay. Data were expressed as the mean ± SEM from at least three separate experiment (* *p* < 0.05).

U0126, a pharmacologic inhibitor, can block MEK1/2-mediated phosphorylation of ERK1/2 [44]. In this study, U0126 was analyzed for its effect on vaccinia virus replication. As shown in Figure 5, U0126 completely suppressed the replication of oncoVV-AVL to the level of oncoVV, which was totally different from an upregulation effect of U0126 on oncoVV, as shown in our data. The results indicated that the oncoVV replication upregulated by AVL was completely dependent on ERK activation.

### 2.6. OncoVV-AVL Has Significant Antitumor Activity in Mice

**Figure 6:**
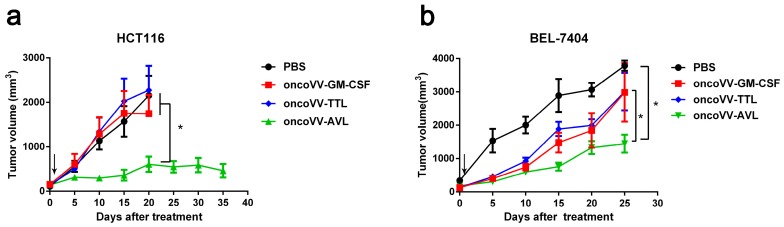
The antitumor effect of oncoVV-AVL on BEL-7404 and HCT116 tumors. (**a**) HCT116 cells or (**b**) BEL7404 cells were injected into the Balb/c nude mice on the back. Tumors were then injected with PBS, oncoVV-GM-CSF, oncoVV-TTL, or oncoVV-AVL. Arrows indicate injections. Data were expressed as the mean ± SEM. (* *p* < 0.05).

To assess the efficacy of oncoVV-AVL against tumors in vivo, subcutaneous tumors were established in Balb/c nude mice with BEL-7404 and HCT116 cells. Tumors were then treated with PBS, oncoVV-TTL [21], oncoVV-GM-CSF, as well as oncoVV-AVL. As shown in Figure 6a, oncoVV-TTL and oncoVV-GM-CSF did not significantly suppress HCT116 tumor growth, as compared to PBS control, while oncoVV-AVL significantly inhibited the growth of HCT116 tumors in mice. As shown in Figure 6b, oncoVV-AVL elicited better antitumor effects on BEL-7404 tumors, as compared with oncoVV-TTL and oncoVV-GM-CSF. Therefore, our data demonstrated that oncoVV-AVL achieved significant therapeutic effect on in vivo tumor models.

## 3. Discussion

Oncolytic vaccinia virus has the advantages of high expression efficiency, low cost, and good oncolytic effect [45]. Human kind has accumulated abundant clinical experience with vaccinia virus due to its successful use in eliminating smallpox. Since the end of the last century, researchers have explored recombinant VV and other poxviruses as expression vectors to trigger immunization in cancer [46,47]. In this study, oncolytic vaccinia virus harboring the marine lectin AVL gene was evaluated for its anti-tumor effects. Our results showed that AVL significantly enhanced the replication of oncolytic vaccinia virus and improved the antiproliferative efficacy of oncolytic vaccinia virus in various tumor cells lines. Importantly, the in vivo antitumor efficacy of oncoVV-AVL was significantly better than that of oncoVV-GM-CSF and oncoVV-TTL. Our data suggested that oncoVV-AVL may be further developed to be an anticancer agent. However, due to isolated AVL being unavailable in our laboratory at present, we are unable to determine whether isolated AVL affects cancer cells with or without the presence of oncoVV. Furthermore, it is still impossible for us to draw a conclusion that oncoVV-AVL has advantage over isolated AVL in treating cancers.

As reported previously, ERK is required for vaccinia virus replication [43]. In this study, AVL stimulated ERK phosphorylation. Furthermore, U0126 inhibitor analysis showed that AVL upregulated oncoVV replication completely dependent on ERK activation. Interestingly, in our previous study, *Tachypleus tridentatus* plasma lectin TTL was also shown to enhance oncoVV replication in an ERK activation dependent manner [21]. Therefore, we propose here that ERK may serve as a common target for a variety of lectins, pending further investigations.

## 4. Materials and Methods

### 4.1. Cell Culture

Human embryonic kidney cells HEK293A, U87 glioma cells, colorectal cancer cells HCT116, human colon cancer cells HT-29, Firefly luciferase-labeled mouse breast cancer cells 4T1-LUC, U251 glioma cells, and hepatocellular carcinoma cells MHCC97-H and BEL-7404 were provided by American Type Culture Collection (Rockville, MD, USA). Cells were cultured in a DMEM medium (Gibco, Thermo Fisher Scientific, Waltham, MA, USA) with 10% fetal bovine serum and 1% Penicillin-Streptomycin Solution.

### 4.2. Generation of OncoVV-AVL

*Aphrocallistes vastus* lectin (AVL, GenBank accession No. AJ276450.1) gene was purchased from Shanghai Generay Biotech Co., Ltd., Shanghai, China. Then, the Flag-AVL gene was inserted into the plasmid pCB with Thymidine kinase (TK) gene deletion. After HEK-293A cells were infected with wild type vaccinia virus (Western Reverse) about 2–4 h, pCB-Flag-AVL was transfection into 293A cells. Vaccinia virus oncoVV-AVL was obtained after 48 hrs. Mycophenolic acid, dioxopurine, and hypoxanthine were added to screen effective oncoVV-AVL. Recombinant viruses were gathered from cell culture medium. The virus titers were determined by TCID_50_ (median tissue culture infective dose).

### 4.3. Cytotoxicity Detection and Flow Cytometry Assay

Cells were seeded in 96-well plates at 5 × 10^3^ per well one day before infection with viruses. Cells were then infected with viruses (Ad-EGFP, Ad-AVL, oncoVV, or oncoVV-AVL), at corresponding multiplicity of infections (MOIs), for the time period as indicated. The cell viability was determined by 3-(4,5-dimethylthiazol-2-yl)-2,5-diphenyltetrazolium bromide (MTT) assay [48]. Meanwhile, cells were infected with oncolytic vaccinia virus at 2MOI for 24 h. Cells were then collected and stained with Annexin V-FITC Apoptosis Detection Kit I (BD Biosciences, San Jose, CA, USA) following the manufacturer’s instruction, and analyzed under an ACEA NovoCyte flow cytometry (ACEA Biosciences, San Diego, CA, USA).

### 4.4. Virus Replication Assay

To determine the viral replication capacity in multiplicity of cells line, cells were plated on 24-well plates at 5 × 10^4^ cells per well one day before treatment with viruses [21]. Cells were infected with oncoVV, oncoVV-GM-CSF or oncoVV-AVL at 5MOI for 2 h, 12 h, 24 h, and 36 h. 10 μM of inhibitor U0126 was used for combination. Cells and culture medium were then collected in a −80 °C refrigerator. The viral titers were determined through tissue culture infectious dose (TCID_50_) assay on 293A cells after three cycles of freeze-thaw in −80 °C and 37 °C.

### 4.5. Animal Experiments

Female Balb/c nude mice (Shanghai Slack Animal Laboratory, Shanghai, China) of 4–5 weeks ages were used for hepatocellular carcinoma and colorectal cancer tumor bearing mouse models. BEL-7404 and HCT116 cells were injected subcutaneously at 5 × 10^6^ cells/mouse and at 8 × 10^6^ cells/mouse respectively into the mice on the back. Mice were divided into groups as indicated at 6–8 mice per group. When tumor size reached a certain volume, oncolytic vaccinia viruses were injected into mice intratumorally at 1 × 10^7^ plaque-forming units (PFU) each. Then, the volume of tumors was measured every five days. The tumor volume was calculated using the formula: length (mm) × width (mm)^2^ × 0.5. Mice were cared for in accordance with the Guide for the Care and Use of Laboratory Animals (Zhejiang Sci-Tech University).

### 4.6. Western Blotting

The cell extracts were subjected to SDS-PAGE and electroblotted onto nitrocellulose membranes. Then, the membranes were blocked with Tris-buffered saline and Tween -20 containing 5% of bovine serum albumin at room temperature for 2 h [49], following incubation with corresponding antibodies overnight at 4 °C. The membranes were washed and incubated with appropriate dilution of secondary antibodies for 1 h at room temperature. After washing with Tris-buffered saline, the bands were detected under a Tanon 5500 chemiluminescence image system (Tanon Inc., Shanghai, China).

Rabbit anti-MDA5 and mouse anti-Bax antibodies were purchased from Santa Cruz Biotechnology Inc. (Dallas, TX, USA). Rabbit anti-ERK1/2, phospho-ERK1/2, rabbit anti-caspase-3, rabbit anti-caspase-8, rabbit anti-NF-κB2, phosphor-NF-κB2, and rabbit anti-GAPDH antibodies were purchased from Cell Signaling Technology Inc. (Danvers, MA, USA). Rabbit anti-Flag was purchased from Bioss Antibodies (Beijing, China). The HRP conjugated goat anti-rabbit and goat anti-mouse were purchased from MultiSciences (Lianke) Biotech Co., Ltd. (Hangzhou, China).

### 4.7. Statistical Analysis

Differences among the different treatment groups were determined by student’s t-test. *p* < 0.05 and was considered significant.

## 5. Conclusions

Our studies showed that oncoVV-AVL elicited significant antitumor activity in a hepatocellular carcinoma as well as colorectal cancer mouse models. AVL enhanced viral replication in an ERK activity dependent manner. Our studies might provide insights into the utilization of AVL in oncolytic viral therapies.

## Supplementary Materials

The following are available online at https://www.mdpi.com/1660-3397/17/6/363/s1, Figure S1: Densitometry analysis of western blots in HCT116 cells.
