# Supplementary material for: Oncolytic Vaccinia Virus Expressing Aphrocallistes vastus Lectin as a Cancer Therapeutic Agent
Source: Mar Drugs. 2019 Jun 19;17(6):363. doi: 10.3390/md17060363 (PMC6628141; doi:10.3390/md17060363)
Supplement: Supplementary file 1 [file marinedrugs-17-00363-s001.pdf]

marine drugs

Oncolytic Vaccinia Virus Expressing *Aphrocallistes vastus* Lectin as a Cancer Therapeutic Agent

Tao Wu, Yulin Xiang, Tingting Liu, Xue Wang, Xiaoyuan Ren, Ting Ye*, Gongchu Li*

College of Life Sciences and medicine, Zhejiang Sci-Tech University, Hangzhou 310018, China; wutao0920@163.com (T.W.); q522329467@163.com (Y.X); m13617965853@163.com (T.L); wx18815610822@163.com (X. W.) imrenxy@163.com (X.R.)

Correspondence: lgc@zstu.edu.cn (G.L.); yeting@zstu.edu.cn (T.Y.)

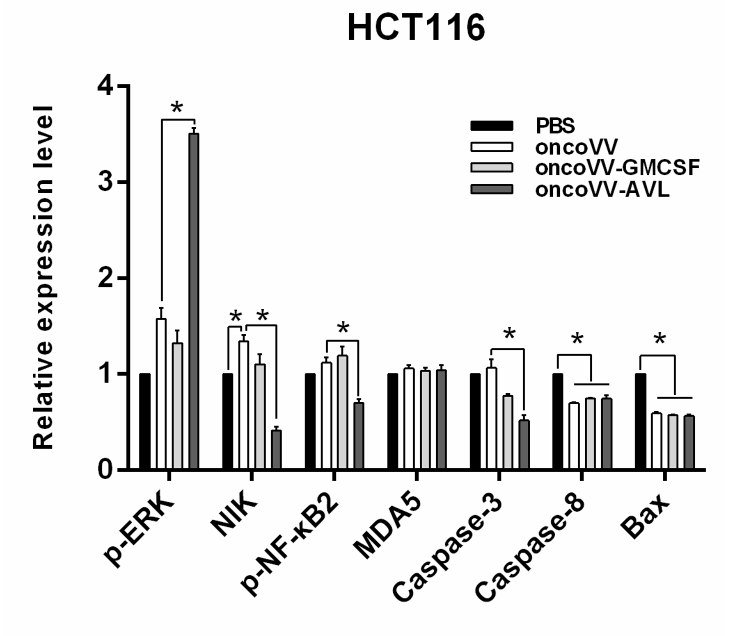

Supplementary Figure 1. Densitometry analysis of western blots in HCT116 cells. Western blot quantification was performed by the ImageJ software. Relative phosphorylation levels of NF-κB2 and ERK were calculated as the ratio to total NF-κB2 and ERK. Relative levels of caspase 8, Bax, caspase 3, MDA5, and NIK were calculated as the ratio to GAPDH.
